# Brainstem evoked response audiometry in normal hearing subjects

**DOI:** 10.1016/S1808-8694(15)30661-3

**Published:** 2015-10-19

**Authors:** Maria Carolina Braga Norte Esteves, Ana Helena Bannwart Dell' Aringa, Gustavo Viani Arruda, Alfredo Rafael Dell' Aringa, José Carlos Nardi

**Affiliations:** 1 Medical graduate, third-year otorhinolaryngology resident; 2 Master's degree student, FMRP. Speech therapist of the Otorhinolaryngology Discipline, Faculdade de Medicina de Marilia; 3 Medical course, radiotherapist, Faculdade de Medicina de Marilia; 4 Doctorate, head of the Otorhinolaryngology Discipline, Faculdade de Medicina de Marilia; 5 Master's degree, assistant professor of the Otorhinolaryngology Discipline, Faculdade de Medicina de Marilia

**Keywords:** audiometry evoked response, literature, standards

## Abstract

Brainstem Evoked Response Audiometry (BERA) is an objective and non-invasive method of hearing assessment which detects electrical activity from the inner ear to the inferior colliculus.

**Aim:**

To assess the hearing pathway in normal hearing individuals and compare differences associated with gender, age and ear side (left and right). Study Design: A retrospective study.

**Materials and Methods:**

Sixty normal hearing individuals, aged between 09 and 66 years old, were subjected to clinical ENT examination and audiologic tests.

**Results:**

Wave latencies differed significantly between males and females, although there were no differences regarding right or left ear sides. Comparing latency averages regarding age and gender we noticed important differences. By the same token, significant differences were also seen comparing this study with the information present in the handbook of the BERA device used and results published by Fukuda, in another study.

**Conclusion:**

Knowing the great importance of BERA, it is crucial that each service develops its own standards in order to enhance the accuracy of the electrophysiological diagnosis of the hearing pathway.

## INTRODUCTION

The brainstem auditory evoked potential (BAEP) is an objective electrophysiological method for assessing the auditory pathways from the auditory nerve to the brainstem. It is considered a short latency potential, since it occurs within the first 10 milliseconds after a sound stimulus is presented.

The BAEP comprises seven waves, of which waves I, III and V are the most visible and of more significant clinical value. The currently used classification for the generating site of these waves is: I - distal portion of the auditory nerve relative to the brainstem; II - proximal portion of the auditory nerve relative to the brainstem; III - cochlear nucleus; IV - superior olivary complex; V - lateral lemniscus; VI - inferior colliculus; and VII - medial geniculate body.^1^,^2^

Recordings of this potential may be clinically analyzed according to a number of parameters: morphology; absolute latency and wave I, III and V amplitude; I-III, I-V and III-V interpeak interval latencies; I-V latency and amplitude relation; and I-V interval interaural difference or wave V absolute latency difference.3 Absolute latency and interpeak interval measurements are those most widely used clinically.

According to the literature, the main clinical aims of BAEP are: to establish a minimal auditory response level, to characterize the type of hearing loss, to assess the maturity of the central auditory system in neonates, to define the site of auditory nerve or brainstem injury, to monitor surgery of the posterior fossa, and to monitor patients in intensive care units.^3^, ^4^, ^5^

Many authors have investigated the interference caused by physiological factors on BAEP recordings. These consist of subject-related features, such as age, sex and hormonal status. In some studies,^6^, ^7^, ^8^, ^9^ increased wave latencies has been observed in subjects aged over 60 years, while others have demonstrated no statistically significant differences in BAEP latency with age.^5^,^10^,^11^ Other studies have shown that latency measures (especially wave V) and interpeak intervals (especially in the I-V interval) are higher in male subjects compared with female subjects.^12^, ^13^, ^14^ Thus, both age and sex are mentioned as variables that may alter BAEP recordings; their true influence, however, remains controversial, requiring additional studies of these issues.

Therefore, the purpose of this study was to analyze absolute wave I, III and V latencies and I-III, III-V and I-V interpeak intervals in audiologically normal subjects of both sexes.

## MATERIAL AND METHOD

The Otorhinolaryngology Discipline of a medical school conducted this study; it was approved by the Research Ethics Committee (n° 392/07).

Within a 12-month period, 60 patients aged from 9 to 66 years (mean = 37.26 years) were selected; there were 21 male and 39 female subjects. All patients with auditory and/or vestibular complaints were seen at the otorhinolaryngology unit, and underwent an otorhinolaryngological exam (otoscopy), audiological studies (pure tone audiometry, immittance testing, distortion product evoked otoacoustic emissions) and an electrophysiological assessment (brainstem auditory evoke potential, or BAEP).

Inclusion criteria were: a normal otoscopy; pure tone audiometry thresholds equal to or below 20 dB at 250 Hz, 500 Hz, 1 KHz, 2 KHz, 3 KHZ, 4 KHz, 6 KHz and 8 KHz; normal immittance test with the presence of the ipsilateral and contralateral stapedial reflex; and distortion product evoked otoacoustic emissions from 328 to 6703 Hz in both ears. Exclusion criteria were: any of the abovementioned test with altered results (not within normal limits); and patients with suspected or confirmed neurological diseases, since these conditions may yield normal audiological tests and altered auditory evoked potentials.

BAEP were recorded with an Intelligent Hearing Smart EP two-channel device with four disposable electrodes; two were placed on the frontal area (ground and positive electrodes) and one in each mastoid (negative electrodes). An alternate polarity click was used as the acoustic stimulus; 19 clicks per second were delivered through monaural insertion earphones at 80 dB nHL, totaling 2,048 stimuli.

Data were gathered for a horizontal retrospective study and patient file analysis, which characterized a cross-sectional historical cohort study. Absolute values (in milliseconds) of absolute latencies and wave I, III and V interpeak intervals were analyzed for each ear. The exam measurement means were calculated according to sex, the side (right or left side) and age of patients to analyze possible differences among absolute wave latency period values. Sex and age measures were compared with other published studies. Student's T test for single independent samples was applied for the analysis of absolute value and latency period means. Levene's test was applied for analyzing the equality of variances. Statistically significant values were those below p < 0.05. The SPSS 15.0 software was used for these tests. This study was designed to detect a 0.05 ms difference among absolute latency measures and wave interpeak intervals; the statistical power was 80% and the significance level was 5%.

## RESULTS

Table 1 shows the absolute latency value and the wave I, III and V interpeak interval means with their standard deviation (SD) in 120 ears regardless of sex or side.

**Table 1:** Mean and standard deviation (ms) of absolute latencies (n = 120 ears).

	Wave	Wave	Wave	Interval	Interval	Interval
	I	III	V	I - III	III - V	I - V
Mean	1,69	3,82	5,59	2,13	1,78	3,90
Standard	±	±	±	± 0,14	± 0,18	± 0,21
deviation	0,13	0,16	0,20			

**Chart 1:**
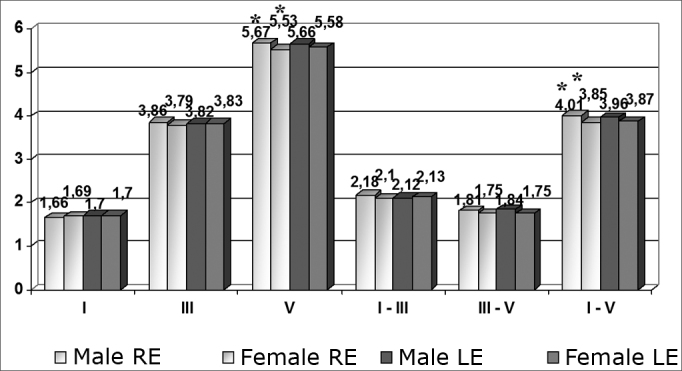
Comparison between sexes according to the ear (n = 120 ears) - * = p < 0.05

Comparing male and female patients according to the ear (right ear - RE; left ear - LE), statistically significant differences were found only in wave V and the interval I-V in the right ear. Other mean differences were not statistically significant (Chart 1 and Table 2 which show standard deviations for these means and p values).

**Table 2:** Mean, standard deviation (ms) and p value according to sex (n= 120 ears).

	Male RE	Fem RE	Value p	Male LE	Fem LE	p Value
Wave I	1.66± 0,13	1.69± 0,13	0.841	1.7± 0,16	1.7± 0,12	0.80
Wave III	3.86± 0,16	3.79± 0,15	0.992	3.82± 0,16	3.83± 0,16	0.996
Wave V	5.67± 0,25	5.53± 0,17	0.047	5.66± 0,21	5.58± 0,18	0.537
Wave I-III	2.18± 0,15	2.1± 0,16	0.723	2.12± 0,13	2.13± 0,18	0.580
Wave III-V	1.81± 0,19	1.75± 0,17	0.447	1.84± 0,15	1.75± 0,15	0.937
Onda I-V	4.01± 0,29	3.85± 0,15	0.007	3.96± 0,22	3.97± 0,19	0.571

Results of comparing between right and left ears regardless of sex were not statistically significant. The means according to age, sex and ear, separating right and left ears in male and females patients below and above age 35 years were compared. There was only a single significant change found when comparing intervals I-V in the right ears of female patients below and above age 35 years (p = 0.000), as shown in Table 3.

**Table 3:** Right ear means (ms) in females and p value (n= 39 ears).

Females	I	III	V	I-III	III-V	I-V
RE < 35 a	1,72	3,80	5,53	2,08	1,73	3,80
RE > 35 a	1,69	3,85	5,61	2,15	1,76	3,92
p Value	0,30	0,36	0,12	0,17	0,38	0,00

**Chart 2:**
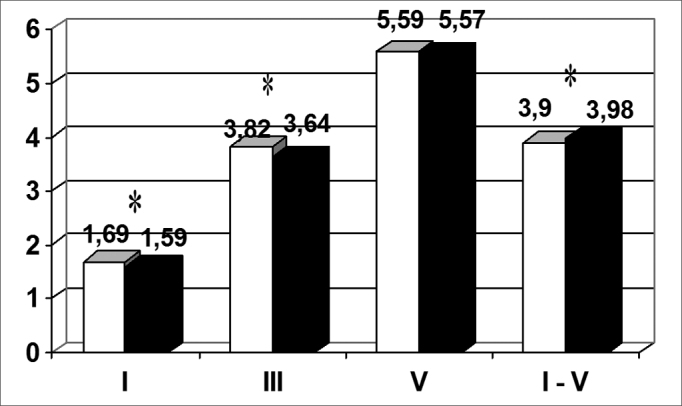
Comparison between the means found and means suggested by the manual of the device (n = 60 patients) - * = p < 0.05

There were statistically significant differences (p < 0.05) between wave I, III and interval I-V means in this study and the means shown in the manual of the device, extracted from a paper by Jacobson et al. (1985), as shown in Chart 2. Other means are also shown in this Chart.

Table 4 shows the differences among wave I, III and V absolute latency and interpeak interval I-V means found in this study and those suggested by Jacobson et al.; p values for these comparisons and 95% confidence intervals (CI) are also shown.

**Table 4:** Difference among absolute latency means (ms), the p value and the confidence interval (CI) (n = 60 patients)

	Wave I	Wave III	Wave V	Interval I - V
Difference among means	0,10	0,18	0,02	0,08
p value	0,000	0,000	0,209	0,000
CI (95%)	0,08 – 0,13	0,15 – 0,21	-0,01 – 0,06	-0,11 - −0,04

**Chart 3:**
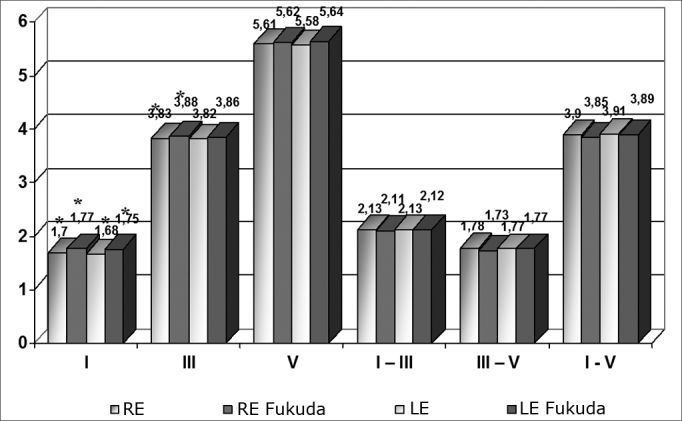
Comparison between the means found and means suggested by Fukuda et al.'s study (n = 120 ears) - * = p < 0.05

The means obtained in this study were also compared with those from a study by Fukuda and cols. (1988).^15^ These means were separated according to the right or left ear, regardless of sex. The analysis revealed a significant difference between wave I in both sides, and wave III in the right ear only. Chart 3 shows these means and other that were not relevant.

Table 5 and 6 show differences among wave I, III and V absolute latency and interpeak interval I-III, III-V and I-V means and those found by Fukuda et al., and the p values for these comparisons and the 95% confidence intervals (CI) in the right and left ears.

**Table 5:** Difference among absolute latency means (ms), the p value and the confidence interval (CI) in the right ear (RE) (n= 60 ears).

RE	I	III	V	I - III	III - V	I - V
Difference between means	0,07	0,05	0,01	0,02	0,05	0,05
p value	0,000	0,005	0,147	0,316	0,102	0,056
CI (95%)	-0,12 a −0,05	-0,10 a −0,20	-0,10 a 0,01	-0,02 a 0,06	-0,01 a 0,08	0,00 a 0,11

**Table 6:** Difference among absolute latency means (ms), the p value and the confidence interval (CI) in the left ear (LE) (n= 60 ears).

LE	I	III	V	I - III	III - V	I - V
Difference between means	0,07	0,04	0,06	0,01	0,00	0,02
p value	0,009	0,141	0,194	0,759	0,655	0,631
CI (95%)	-0,08 a −0,01	-0,07 a 0,01	-0,08 a 0,02	-0,04 a 0,05	-0,03 a 0,05	-0,04 a 0,06

## DISCUSSION

The BAEP is an important test in clinical practice; it is used to diagnose auditory threshold changes to characterize the type of hearing loss, to identify retrocochlear or central nervous system alterations, and to assess central auditory system maturity in neonates. Its sensitivity for detecting these conditions is considered optimal, since it does not depend on information from patients.

Because of questions raised by many authors about interferences from certain physiological factors, such as age and sex, on BAEP recordings, a study was needed to assess these variables in normal individuals. As this test becomes more widely used in clinical practice, normatization protocols for each healthcare institution need to be discussed for comparisons with data from other institutions and other studies.

This study presents wave I, III and V absolute latency and interpeak interval I-III, III-V and I-V means gathered from 60 male and female patients aged from 9 to 66 years for comparisons within the sample and with other studies.

The analysis revealed that there was a significant difference in the wave V latency time and the I-V interpeak interval between sexes; latencies were higher in the right ears of males (Chart 1). This result supports other published data,^10^,^12^,^13^,^14^,^16^ which show this wave V and interpeak interval I-V increase in males.

There were no statistically significant differences in comparisons between right and left ears regardless of sex. Fabiano et al.^17^ also reached similar conclusions. No latency differences between ears are expected in patients with bilaterally normal auditory thresholds.

Many papers have demonstrated that BAEP recordings change in elderly subjects. Burkard and Sims (2002)^18^ and Boettcher (2002)^19^ assessed individuals with presbycusis and found that absolute wave latencies in BAEP were increased and that interpeak latencies were unaltered.^18^,^19^ Matas et al.^4^ also found a progressive increase of BAEP recordings with age; in the 70 to 79 year age group, BAEP alterations were found in 85% of ears.^20^ Ottaviani et al.,^7^ Rosenhall et al.,^6^ and Freitas and Oliveira^8^ found that age-related hearing loss (presbycusis) is described in BAEP as increased electrophysiological thresholds, increased latencies and/or decreased wave amplitude in humans and animals. For this reason comparisons between young and elderly patients with presbycusis are not recommended.^7^,^21^,^22^ Beagley and Sheldrake^10^ and Anias et al.^11^ studied normal-hearing subjects and found no age-related effect on absolute latencies; this may be explained by the inclusion criteria of that study, which took into account only normal-hearing ears. According to Anias et al.,^11^ controversies in the literature about the relation between BAEP and age in adults may be due to varying selection criteria, especially those related with the health status, auditory selection criteria, sex and the stimulus unit. Only one statistically significant change was found in our study: the interval I-V was higher in the right ears of female subjects aged over 35 years compared to female subjects aged below 35 years, which supports published results. Thirty-five was the median age, and was thus chosen as the cutoff age.

A statistical analysis of results revealed a significant difference between wave I and III absolute latency times and the interpeak interval I-V values found in this study and values suggested by the equipment manual. Such data demonstrate the need for each institution to standardize its own wave absolute latency and interpeak interval values for each device, regardless of values suggested in the literature, to avoid incorrect diagnoses.

A comparative analysis of our means with those in Fukuda et al.'s 1988 study^15^ revealed a significant difference between wave I (both sides) and wave III (right ear), once again showing that different devices and examiners may yield distinct results; thus, each institution should have its own parameters.

These differences may also be explained by the fact that this study was designed to detect a 0.05 ms difference among means. Statistically significant differences were equal to or higher than 0.05 ms.

A study with a larger sample is required to reduce this value and possibly detect smaller differences.

## CONCLUSION

Considering that the differences found in this study do not yield different medical interpretations, since exams are within normal limits and will therefore not alter subsequent approaches, this study aimed mainly to highlight the importance of each institution to set its own parameters - as shown in the analyses - to avoid controversies in results when compared with other institutions.

Because this exam is important and widely applicable, it is essential for each institution to conduct their own parameter-setting study to increase the accuracy of the electrophysiological assessment of auditory pathways.
